# The Hedonext^®^ Method—A Case Study with Extra Virgin Olive Oil

**DOI:** 10.3390/foods15020276

**Published:** 2026-01-12

**Authors:** Jean-Xavier Guinard, Ellen Mayra Menezes Ayres, Karen Gee, Eleonore Loescher, Jean-Marc Sieffermann

**Affiliations:** 1 Department of Food Science and Technology, University of California, Davis, CA 95616, USA; kmgee@ucdavis.edu; 2 Department of Fundamental Nutrition, School of Nutrition, Federal University of the State of Rio de Janeiro (UNIRIO), Rio de Janeiro 20051-030, Brazil; ellen.menezes@unirio.br; 3 LVMH Recherche, 75008 Paris, France; eloescher@research.lvmh-pc.com; 4 AgroParis Tech, Paris-Saclay University, 91120 Palaiseau, France; jms.agroparistech@yahoo.fr

**Keywords:** Hedonext, 9-point hedonic scale, consumer testing, preference mapping, olive oil

## Abstract

Consumer choice, liking, and preference can be assessed in different ways. We tested the application of a novel method called Hedonext^®^ for the measurement of consumer liking and preference for extra virgin olive oil (EVOO). Consumers were instructed that they would be presented with up to eight olive oils and invited to taste each of them in sequence until they found the oil that they liked or preferred, thus ending the test, and that they would receive that oil as their reward. We compared the outcomes of the Hedonext Method to hedonic ratings on the nine-point hedonic scale of all eight oils, which were then analyzed by preference mapping. A total of 139 and 141 consumers, who consumed EVOO at least once a week and were evenly distributed by age (18–65 years old) and gender (60% women, 40% men), evaluated the set of olive oils from Italy, Spain, or California with Hedonext or the nine-point hedonic scale, respectively. The oils that were chosen most often by consumers in the Hedonext procedure were also the oils that received the highest mean hedonic ratings on the nine-point hedonic scale, and those picked the least often with Hedonext also received the lowest hedonic ratings. Traditional preference mapping from hedonic ratings and an alternative preference mapping approach through Hedonext also produced similar outcomes. The Hedonext Method was shorter to administer and was enjoyed by the consumers who experienced it because of its game-like nature. It does require, though, that the product under testing be perceived as valuable by consumers, as it serves as the reward for their participation. We conclude that the Hedonext Method represents a novel and valuable alternative to the 9-point hedonic scale, particularly for the evaluation of premium or luxury consumer products.

## 1. Introduction

New product development or product optimization requires fast, simple, and affordable methods that reliably capture the essence and context of the consumer experience and predict purchase decisions, future sales, and hence potential success of a product. The product supply is also increasing, with thousands of new consumer products launched yearly on the main world markets (and a large percentage of those failing past their launch year). Consumers are thus confronted with a choice between more and more products. But when buying a new product, they do not necessarily know all the products on the market, nor do they have the possibility or the will to test them all. This abundance of choice, therefore, is something that should be factored into the design of market research and product developer toolboxes [1].

For 75 years now, the most common approach to assessing consumer acceptance of a product or service has been to measure liking on the nine-point hedonic scale [2] or another liking scale, typically in a sequential monadic fashion [3]. And a paired-preference test, sometimes with a no-preference option or preference ranking for multiple products, has been the norm for the assessment of preference [4]. A comparison of the nine-point hedonic scale with the labeled affective magnitude scale and an eleven-point liking category scale showed similar outcomes and discrimination among products across different product categories [5]. Other novel methods for the determination of consumer liking and preference include conjoint analysis [6,7], best-worst scaling [3], and ‘liking’, ‘buying’, ‘choosing’, and ‘take away’ preference tests [8]. Willingness to pay and purchase intent have also been used as alternatives to hedonic ratings [9,10]. It remains that the nine-point hedonic scale, despite its inherent flaws or potential biases, is the most used instrument for the measurement of consumer product acceptance [11]. Just-about-right scaling was introduced more recently to assess the suitability or appropriateness of specific sensory attributes [12,13]. And the efficacy of the main methods for measuring consumer acceptance and preference has also been measured [14]. The Hedonext^®^ Method offers an alternative way of approaching how a consumer feels about a product by assessing whether the product is seen as a good acquisition. The Hedonext^®^ Method goes beyond liking and encompasses worth, value, and satisfaction concepts, among others.

The Hedonext^®^ Method was invented by Prof. Jean-Marc Sieffermann and first tested in the food category at AgroParisTech in 2011. That was followed by the first application of the method to cosmetics [15]. In 2015, LVMH and INRA-AgroParisTech patented the method [16]. With the Hedonext^®^ Method, consumers are told they are going to be presented with a set of up to p products. They are then invited to evaluate them sequentially and are asked if they are satisfied enough with the product they just tested. If they answer “yes”, the test stops, and they receive the product as a gift or reward. If they answer “no”, the test continues until they choose a product or test the last product in the set, which then becomes their reward. Consumers cannot go back and select a product they previously tested and moved past.

We tested the hypothesis that Hedonext^®^ and the nine-point hedonic scale would produce similar outcomes regarding consumer acceptance of commercial extra virgin olive oils with different sensory profiles and quality, thus providing further validation of the Hedonext^®^ Method. The main objective of this research was to compare Hedonext^®^ to a conventional hedonic method. Secondary objectives were to document the experience of the Hedonext^®^ users and to explore a ‘preference mapping’ alternative through Hedonext^®^.

## 2. Materials and Methods

### 2.1. Oil Samples

We evaluated the same 8 commercial extra virgin olive oils with both methods—Hedonext and the 9-point hedonic scale. The oils, of different geographical origins (California, Italy, and Spain) and prices (USD 8 to USD 36, average USD 15), were selected to cover a range of sensory profiles, from ripe fruity to green fruity to bitter and pungent, and including rancid and/or fusty notes for some. They are listed in Table 1.

With each method, 45 mL of olive oil was poured into 120 mL coded plastic souffle cups (Dart Container Corporation, Mason, MI, USA) and capped with a plastic cap no longer than one hour before testing.

### 2.2. Consumers

Regular consumers of olive oil were recruited from the Davis, Woodland, and Sacramento communities in Northern California and screened for their (weekly) consumption of olive oil. Individuals with food allergies or dietary restrictions, or employed by the consumer product industry or a consumer testing agency, were screened out. Demographics and olive oil usage and attitudes of the consumers are provided in the Supplemental Materials.

Consumers were randomly assigned to the Hedonext Method (n = 139) or the 9-point hedonic scale method (n = 141).

### 2.3. Hedonext Method

For the Hedonext Method, consumers were instructed that they would be presented with up to 8 olive oils and invited to taste each of them in sequence until they found the oil that they liked or preferred, thus ending the test, and that the oil they had selected would be their reward for the test.

The order of presentation of the oils was randomized using a Mutually Orthogonal Latin Squares (MOLS) design (block of 112 consumers) so that each oil appeared the same number of times in each position (1–8) and before or after each of the other oils in the design [17]. We should note that the experimental design was inherently incomplete because, with Hedonext, consumers did not have to taste all 8 oils in the design, except if they chose the 8th oil in their set.

Upon selecting their oil, consumers were then asked the question, “What did you think about this tasting protocol?”

Consumers were provided with crackers and still water to rinse and cleanse their palates between samples.

### 2.4. Conventional Hedonic Method

With this method, consumers were asked to indicate their overall degree of liking of the 8 oils presented simultaneously on the 9-point hedonic scale [2]. The order of presentation and palate cleansing between samples followed the same method as in Section 2.3. Upon completing their evaluations, consumers received a bottle of the Cobram Estate Extra Virgin Olive Oil as their reward.

### 2.5. Testing Conditions and Approvals

All evaluations, with both methods, were conducted in the Silverado Vineyards Sensory Theater of the Robert Mondavi Institute for Wine and Food Science at UC Davis, a temperature- and lighting-controlled facility equipped with partitions to separate consumers while testing.

This research was approved by the Institutional Review Board of the University of California, Davis (protocol number 1339810-1).

### 2.6. Expert Evaluation of the Oils

To provide descriptive data in support of an exploration of a ‘preference mapping’ alternative through Hedonext, 18 olive oil experts judging olive oils at the Los Angeles International Olive Oil Competition [18] were asked in a separate session to rate the sensory quality of the 8 olive oils in this study, blind, on a 100-point scale, and to provide descriptive comments regarding the oils in a list-all-that-apply (LATA) procedure.

Experts were provided with crackers, plain yogurt, green apple slices, and still or sparkling water to rinse and cleanse their palates between samples.

### 2.7. Data Analysis

For Hedonext, we tallied the number of selections of each of the oils and the selection positions, from 1st to 8th.

For the 9-point hedonic scale, hedonic ratings of the oils across consumers were analyzed by analysis of variance, multiple mean comparisons (Fisher’s LSD), and preference mapping (PCA of the matrix of hedonic ratings across the oils).

The frequency of use of the various descriptors in the LATA task by the experts across the oils was analyzed by multiple factor analysis (MFA) to produce a sensory map of the oils.

Comments regarding the Hedonext Method were transcribed, quotes were categorized, and a word cloud was generated.

Unless otherwise specified, all analyses were performed on R (Version 4.1.0) using the Agricolae, FactoMineR, and ggplot2 packages.

## 3. Results

### 3.1. Hedonext vs. Nine-Point Hedonic Scale

**Figure 1:**
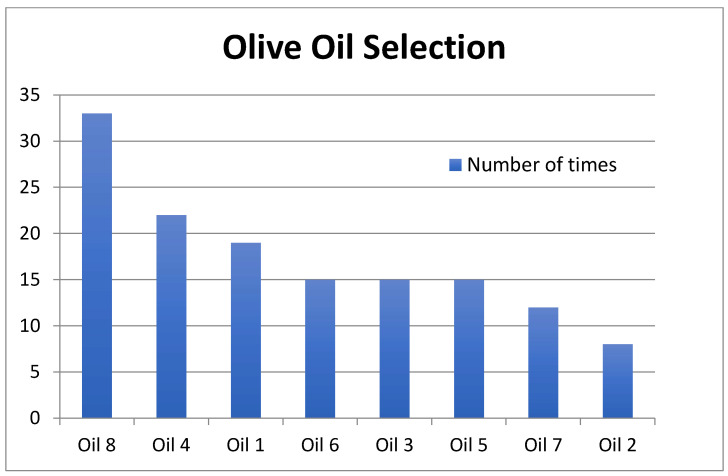
Number of times each oil was selected with the Hedonext method, from highest to lowest, across all eight positions.

Hedonext and the nine-point hedonic scale produced similar results. Figure 1 shows the number of times each oil was selected. Oil 8 was the clear winner with Hedonext, with 33 of the 139 consumers choosing it. It was followed by oils 4 and 1, then a trio of oils—3, 5, and 6—finally oils 7 and 2, with the least numbers of selections at 12 and 8, respectively. When we compare this outcome to the mean hedonic ratings received on the nine-point hedonic scale in Table 2, we can see that oil 8 received the highest mean rating at 6.69, followed by oil 6, and further down by oils 1 and 4, then oils 3 and 2, and finally oils 5 and 7. Those outcomes are therefore comparable, yet there are some slight differences in the ranking of the oils between the two methods.

Consumers took between 5 and 25 min to complete the Hedonext evaluation, whereas the hedonic ratings on the nine-point hedonic scale took between 12 and 25 min.

### 3.2. Hedonext Specifics

**Figure 2:**
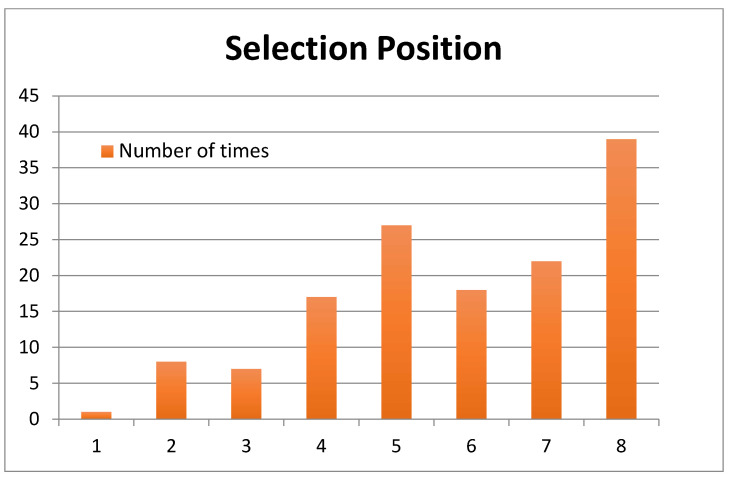
Position of the oils selected with the Hedonext method, for each of the oils, from first position to eighth position.

With regard to the position of the oils that were selected by the consumers with the Hedonext Method, position 8, with 28% of the selections, was the most frequent selection position; that is, the last oil being presented in the set was the most frequent position at which consumers made their selection (Figure 2). The next highest selection was halfway through the set, at position 5.

**Figure 3:**
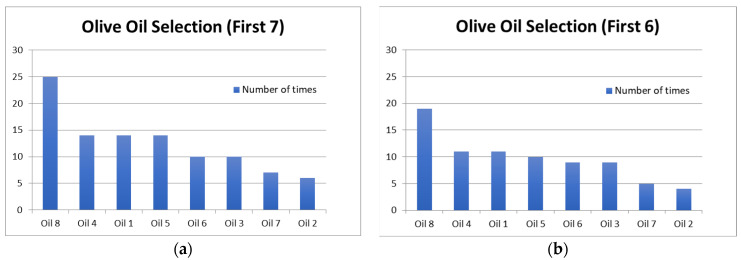
Number of times each oil was selected with the Hedonext method, from highest to lowest, across (**a**) the first seven positions and (**b**) the first six positions.

To ensure that this frequent selection of the last product in the set had not biased the results, we examined the outcomes based on the first 7 or 6 positions only. Those results are shown in Figure 3a,b and they are virtually identical to those of the full set in Figure 2.

**Figure 4:**
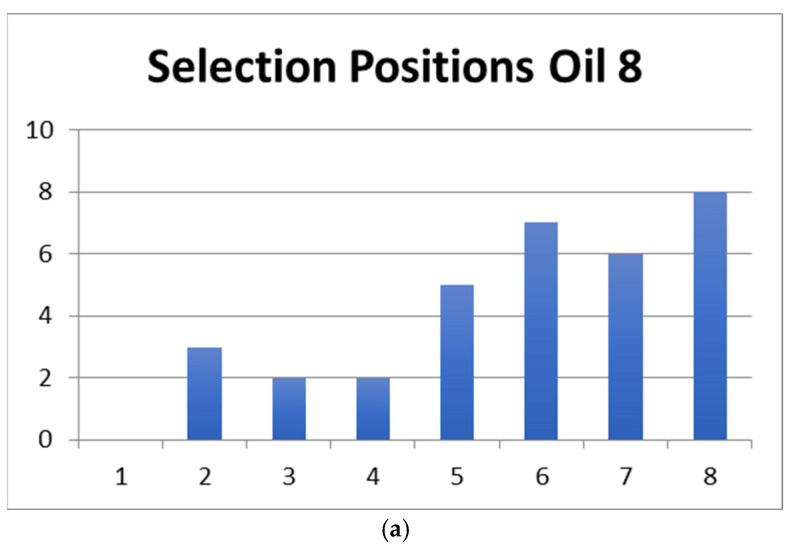

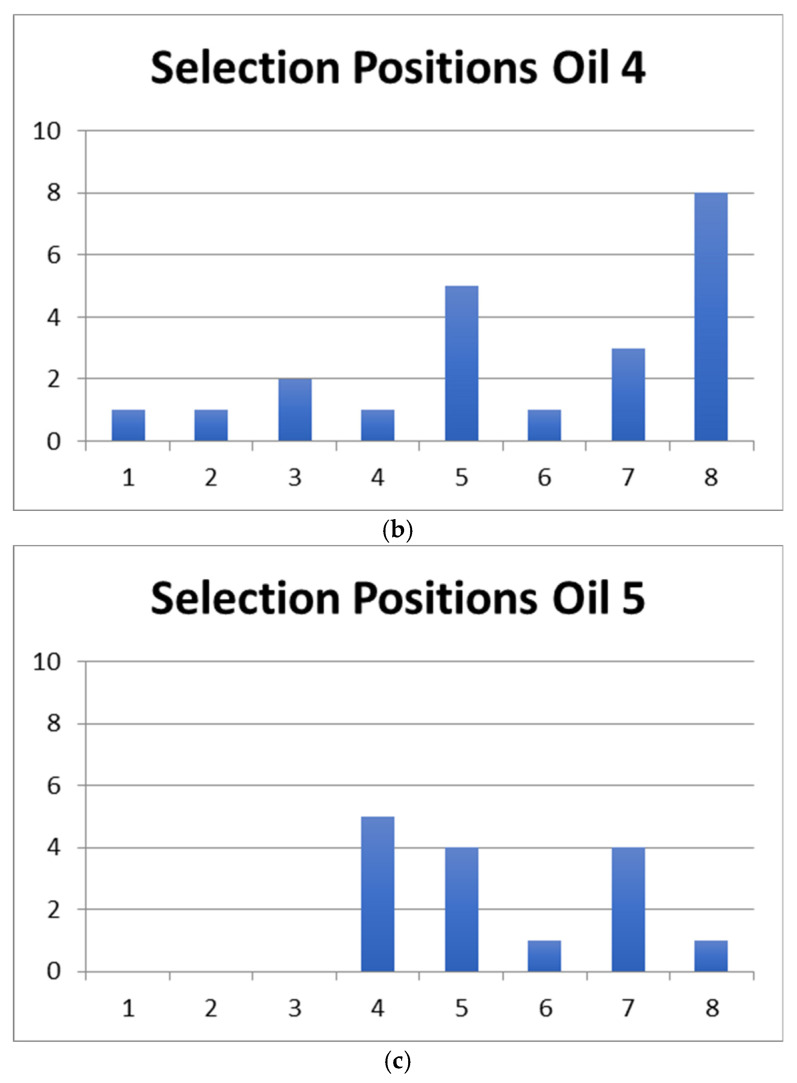
Distribution of selection positions for (**a**) oil 8, (**b**) oil 4, and (**c**) oil 5.

Figure 4, in turn, shows the distribution of selection positions of three oils in the set (most to least often picked), and we find that it closely mirrors that of all the oils combined (Figure 2), except for oil 5, which produced a very different pattern.

**Figure 5:**
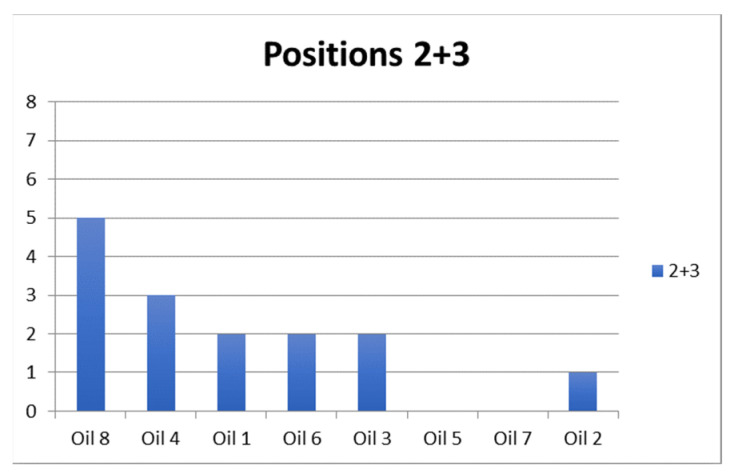

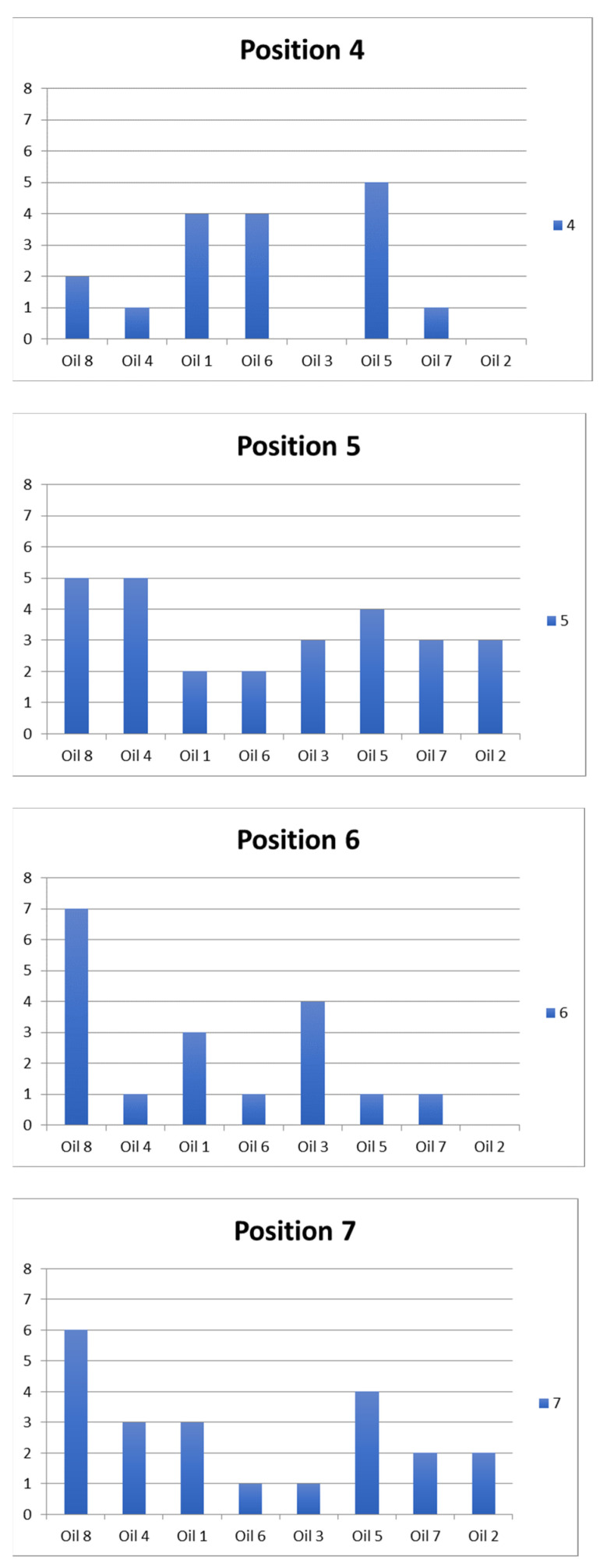
Number of times each oil was selected with the Hedonext method, from highest to lowest overall, for positions 2 and 3, 4, 5, 6, and 7.

And finally, an examination of oil selections by position shown in Figure 5 produced similar outcomes to that for all oils combined (Figure 1), except at position four.

### 3.3. Consumer Feedback on Hedonext Method

**Figure 6:**
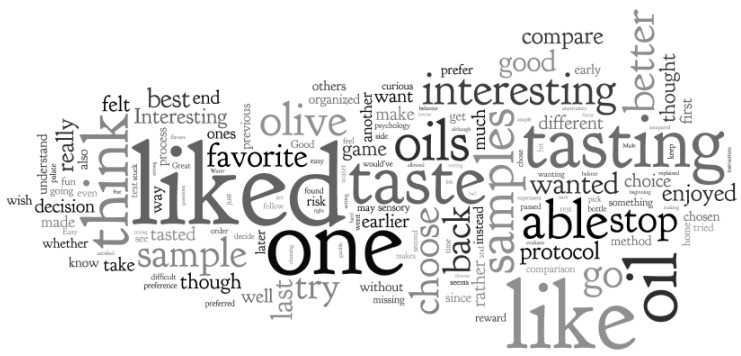
Word cloud derived from the consumers’ open comments regarding the Hedonext method. The larger and bolder the font, the higher the frequency of mention of the word.

Quotes from consumers in answer to the question “What did you think about this tasting protocol?” are compiled in Table 3. And the word cloud derived from those answers is shown in Figure 6. What transpired is that most of the consumers who evaluated the oils with Hedonext liked the method and found it interesting and thought-provoking, but then some consumers were challenged by having to pick just one oil or frustrated from having passed on what had turned out to be their favorite oil in the end, and not being able to go back. The game aspect was highlighted by approximately 20% of consumers as well and appreciated, except if it felt like a gamble, which carried a more negative connotation.

The words ‘like’ and ‘liked’ were mentioned the most, referring to the Hedonext method and/or to the oils, along with other method-specific words such as ‘interesting’, ‘choose’, or ‘stop’. ‘One’ was also mentioned often as a key word in English grammar and in reference to having to choose ‘one’ oil with Hedonext.

### 3.4. ‘Preference Mapping’ with Hedonext?

**Figure 7:**
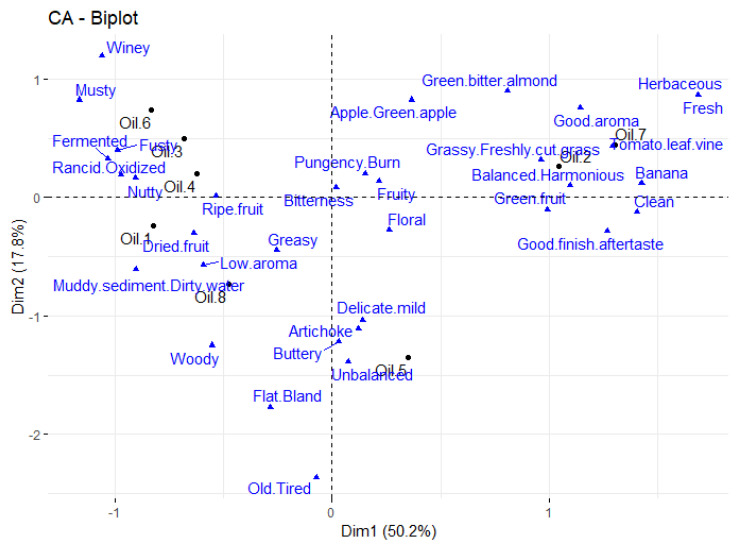
Sensory map of the olive oils derived by multifactor analysis (MFA) of list-all-that-apply (LATA) ratings of the oils by experts from the Los Angeles International Extra Virgin Olive Oil Competition, showing both the oils and the attributes for dimensions 1 and 2.

In order to develop a ‘preference map’ similar to that obtained by means of PCA from a matrix of hedonic ratings of different products, a method based on MFA of expert ratings using the LATA procedure was applied. This facilitated the initial development of a sensory map for the EVOO samples (Figure 7). The first two dimensions in the MFA biplot explained 68% of the sample variability. That biplot groups oils 3, 4, and 6 in the upper left quadrant; oils 1 and 8 in the lower left quadrant; oil 5 by itself in the lower right quadrant; and oils 2 and 7 in the upper right quadrant. It is worth noting that in this biplot, undesirable olive oil attributes are found on the left side of dimension 1, whereas desirable ones are found on the right side. The oil groupings are groupings we can make based on the oil selections of the consumers in Figure 1 as well. And if we assign consumers to those groups based on their oil selections, we end up with 52 consumers in a first ‘preference segment’ (those consumers who picked oils 1 or 8 with Hedonext), 52 consumers in a second ‘preference segment’ (those who picked oils 3, 4, or 6), 15 consumers for oil 5, and finally 20 consumers in a fourth ‘preference segment’ (those who picked oils 2 or 7).

**Figure 8:**
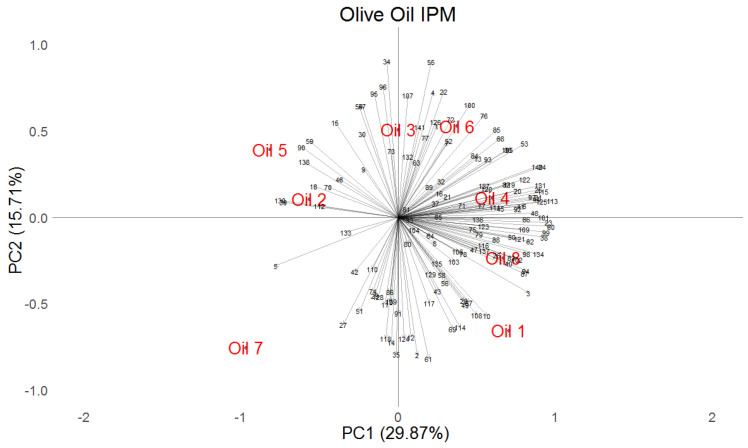
Internal preference map of the matrix of hedonic ratings of the oils on the nine-point hedonic scale by 141 consumers, showing both the consumers (as vectors) and the oils for principal components 1 and 2.

The internal preference map derived from the hedonic ratings on the nine-point hedonic scale in Figure 8 shows a very similar outcome to that of our alternative, Hedonext’s ‘preference map’, with oils 5, 2, and 7 on the left side of the preference map, with relatively few consumer vectors pointing towards those oils, and one large set of consumer vectors or preference segment preferring oils 3, 4, and 6 on the one hand (in the upper right quadrant), and another large set of consumer vectors or preference segment preferring oils 1 and 8 (in the lower right quadrant). If one examines the groupings of the oils, they are consistent between the two figures, regardless of which vectors (sensory attributes in Figure 7 or consumers in Figure 8) they are associated with.

## 4. Discussion

The results obtained with the Hedonext^®^ method (frequency of sample selection) and with the nine-point scale (sample liking scores) were similar. And data collection was faster with Hedonext^®^. Thus, the Hedonext^®^ method is a viable method for measuring consumer acceptance of food or beverages (Figure 1 and Table 2). Yet, the two approaches do not measure the exact same aspect of the consumer’s reception of a product. Hedonext is more reflective of point-of-purchase choice and purchase decisions by the consumer, whereas the nine-point hedonic scale focuses on how the consumer likes the product from a sensory perspective. Regardless, just as the nine-point hedonic scale became the most widely used tool for the assessment of consumer liking of a product because of its ability to predict product success, so could the Hedonext Method become, but it will take the building of a similar database over many products and years.

It should be noted that sample 8 was the most frequently chosen by the Hedonext^®^ method, ahead of samples 4, 1, and 6, in that order. This oil obtained the highest score on the nine-point hedonic scale, but compared to sample 6, it was not liked significantly more. It should also be pointed out that on the nine-point hedonic scale, the order of liking for the samples was reversed, with 6, 1, and 4 being the most to least liked. It could therefore be that Hedonext, with its ‘forced choice’, may be more discriminating than the nine-point hedonic scale.

The position in which consumers made their selection with Hedonext most often was the last one, the 8th position. This is somewhat unusual, as in the original development research for the method with cosmetics, a median position often was the most frequent selection position [14]. In this research, the second most used selection position was the 5th one, indeed. We attribute this to the food nature of the product, which meant only a quick (tasting) interaction with the product, instead of a more extensive and complex experience, as would be the case with cosmetics. Regardless, when we explored what would happen if only the first 7 or first 6 positions were considered (Figure 4a,b), the results were the same as when all 8 positions were tallied, thus confirming the accuracy of the full results, despite this unusually high selection in the last position. We also considered how each oil fared in terms of selection position and found that it mirrored the average (across all oils) distribution in Figure 2 for all oils except oil 5 (Figure 5). The consistency of the ranking of the oils, whether examined by position or by oil (Figure 3a,b, Figure 4, and Figure 5), serves as another validation of the Hedonext Method.

Given the success of our ‘preference mapping’ adaptation to Hedonext through the use of a list-all-that-apply (LATA) task with experts and subsequent multifactor analysis (MFA) of those selections to produce a sensory map of the products, we would suggest incorporating a check-all-that-apply (CATA) task into the Hedonext protocol so that correspondence analysis of those CATA selections could be used to produce a sensory map of the products, onto which the ‘clusters’ of consumers with different choices could be characterized in terms of their respective positive and negative sensory drivers of choice.

When we designed this study, we elected to have two separate populations of consumers with the same number of consumers (i.e., 139 vs. 141) and the same demographic and olive oil usage characteristics (Supplementary Figure S1) evaluate the oils with Hedonext or the nine-point hedonic scale, thus legitimizing the comparison between their outcomes. Indeed, we felt that if the same population had been used to compare the two methods, the first evaluation with one method would potentially have biased the second evaluation with the other method, even if the testing order had been randomized.

One caveat of the Hedonext Method is that it can only be used with products that have a significant reward value—somewhere between low-cost and high-cost items, such as extra virgin olive oil in this study; otherwise, taking home the product one selected may not serve as a substantial enough incentive. This also begs the question of whether the method should best be used with branded products (rather than blinded, as in this study) to best mimic actual situations of choice and purchase.

Another (minor) limitation is that a completely balanced design and full randomization of the order of presentation of the samples (regardless of the MOLS design we used to set up both the Hedonext and the nine-point hedonic evaluations) are not possible with the Hedonext method, because most consumers do not receive the chance to evaluate the full set of samples in the design.

The purpose of this research was to compare the Hedonext Method to a conventional consumer testing method, but if we turn our attention to the olive oils in this design, it is worth noting that there was a significant negative correlation between the consumers’ hedonic ratings of the oils and the quality ratings of those oils by the experts. This is consistent with previous research [19,20,21] and likely was due to most consumers not expecting and/or disliking oils that were bitter and pungent, or their being familiar with and therefore liking those somewhat rancid and fusty oils that still prevail on world markets. Indeed, we and others demonstrated long ago that, unlike taste and chemesthetic preferences, which are mostly innate, olfactory preferences are mostly learned, based on exposure [22].

Potential areas for future research on Hedonext include its respective applicability to central location tests versus home-use tests and the development of a power analysis for Hedonext—how many consumers (n) are required for a set of p products? Finally, there will be a need to further research which statistical tools could be applied to Hedonext data to establish the significance of differences among products and among consumers.

## 5. Conclusions

The two consumer testing methods we evaluated in this study generated similar outcomes; therefore, the Hedonext^®^ Method is a viable alternative to the nine-point hedonic scale for the measurement of consumer acceptance of a product. The oils that were chosen most often by consumers in the Hedonext procedure were also the oils that received the highest mean hedonic ratings on the nine-point hedonic scale, and those picked the least often with Hedonext also received the lowest hedonic ratings. Data collection time was also shorter with Hedonext. Conventional preference mapping from hedonic ratings and an alternative preference mapping approach through Hedonext and expert LATA selections also produced similar outcomes. With 130+ consumers of the same demographic and olive oil usage profiles evaluating the oils with each method, there was ample statistical power to validate our comparison of the two methods. As was anticipated, we found that some consumers enjoyed the novelty and gaming aspects of the Hedonext method, whereas others found it frustrating as they felt they did not end up picking the oil that they liked best.

We look forward to having researchers in academia and the private sector experiment with Hedonext and share the results of their studies with the sensory and consumer science community.

## Figures and Tables

**Table 1:** Extra virgin olive oil (EVOO) samples used in the study, showing brand name (in alphabetical order), geographical origin, volume, and price.

EVOO Brand	Origin	Volume	Price
Bertolli	Italian Blend	500 mL	$8
California Olive Ranch	California	750 mL	$15
Castillo de Canena	Spain	500 mL	$36
Colavita	Italian Blend	500 mL	$10
McEvoy	California	375 mL	$32
Cobram Estate	California	750 mL	$13
Kirkland (Costco)	Italian Blend	2 L	$15
Villa Campestri	Italy	250 mL	$25

**Table 2:** Hedonic ratings of the eight oils on the nine-point hedonic scale, from highest to lowest.

Oil	Mean Degree of Liking and LSD-Derived Superscripts ^1^
8	6.69 ^a^
6	6.37 ^ab^
1	6.14 ^b^
4	6.01 ^b^
3	5.61 ^c^
2	5.23 ^cd^
5	5.04 ^d^
7	4.90 ^d^

^1^ Means sharing superscripts are not significantly different (*p* < 0.05).

**Table 3:** Select quotes on Hedonext method—positives, negatives, and ‘game’ aspect.

**Consumer**	**Positives Quotes**
C6	“It was interesting because I had to take a gamble between if I wanted to try another, or stick to the one I liked.”
C17	“It felt like a psychology test. I really wanted to keep tasting but I didn’t want to bypass really delicious oils. I ended up going for one that really stuck out.”
C44	“It was interesting to have to make a decision on whether or not to continue, without tasting all the samples. I had to think critically about how much I liked the oil.”
C72	“I feel strong feelings of regret. It is a hard balance between wanting to choose one and sticking with it rather than seeing the rest.”
C83	“I felt like stopping at a favorite made me more critical.”
C116	“I liked it! It made me really think about each choice and evaluate how I liked the flavors independent of the samples. Made me think about my preferences versus sample to sample. Interesting protocol!”
C121	“Interesting experience—the element of risk adds to it, makes the decision more high stakes which forces you to think about it more.”
**Consumer**	**Negative Quotes**
C43	“I was surprised that the intention of the tasting was not to try all the different types.”
C57	“Great! Except it made you curious to taste them all instead of stopping.”
C58	“It was interesting to do this in a sequence and not be able to taste all of the samples before choosing my favorite. Perhaps I would have preferred a later sample but did not want to risk having passed my favorite.”
C64	“I felt like part of me was trying to outsmart the test. I felt more like choosing which I could live with rather than which I truly liked.”
C74	“Risk averse. I didn’t want to make it to the last sample only to be disappointed.”
C100	“It was stressful that I could not go back—at the beginning I did not know which one I would like better.”
C128	“Super interesting. I didn’t want to stop right away even though I tried an oil I liked a lot at #2. I was worried the whole time I wouldn’t like anything else.”
C28	“I would prefer being able to go back and do a second round of tasting, so that oils could be compared one against another and the absolute best could be determined.”
**Consumer**	**‘Game’ Aspect Quotes**
C46	“It is interesting. Reminds me of a TV game show.”
C52	“The protocol is like a game show—risk/reward equation. Odd given the premise which I (mis)understood to be giving opinions about olive oils.”
C66	“Unorthodox? Very unsure if this is the best I would like or if I passed up on a bottle I would like. It was like a game to me.”
C79	“It was a bit of a gamble though, since I was unsure how I would feel about future samples and knew I would get to take one home.”
C95	“It was fine. Had the extra catch that you can’t go back to like a previous one after you have moved on which made it a bit of a guessing game if I would like a later one better than the current one. I would have liked a side-by-side comparison to better evaluate them.”

## Data Availability

The original contributions presented in the study are included in the article/Supplementary Materials, further inquiries can be directed to the corresponding author.

## Supplementary Materials

The following supporting information can be downloaded at: https://www.mdpi.com/article/10.3390/foods15020276/s1, Figure S1: Distribution of responses to exit survey questions regarding demographics and olive oil usage for the 9-point hedonic scale consumers (n = 141) and the Hedonext consumers (n = 139).
