# Supplementary material for: The Hedonext® Method—A Case Study with Extra Virgin Olive Oil
Source: Foods. 2026 Jan 12;15(2):276. doi: 10.3390/foods15020276 (PMC12840397; doi:10.3390/foods15020276)

Supplemental Figure S1. Distribution of responses to exit survey questions regarding demographics and olive oil usage for the 9-point hedonic scale consumers (n=141) and the Hedonext consumers (n=139).

a. Age

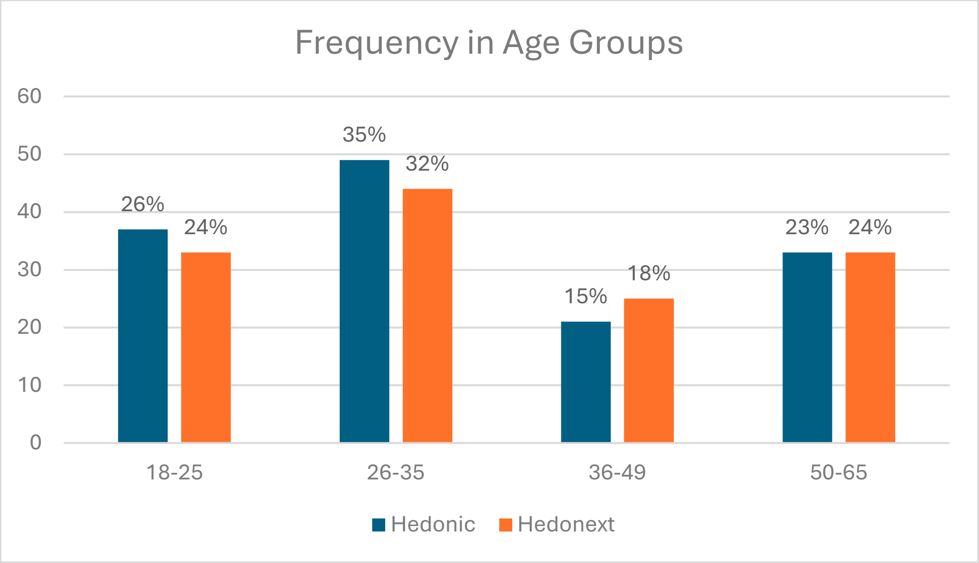

b. Gender

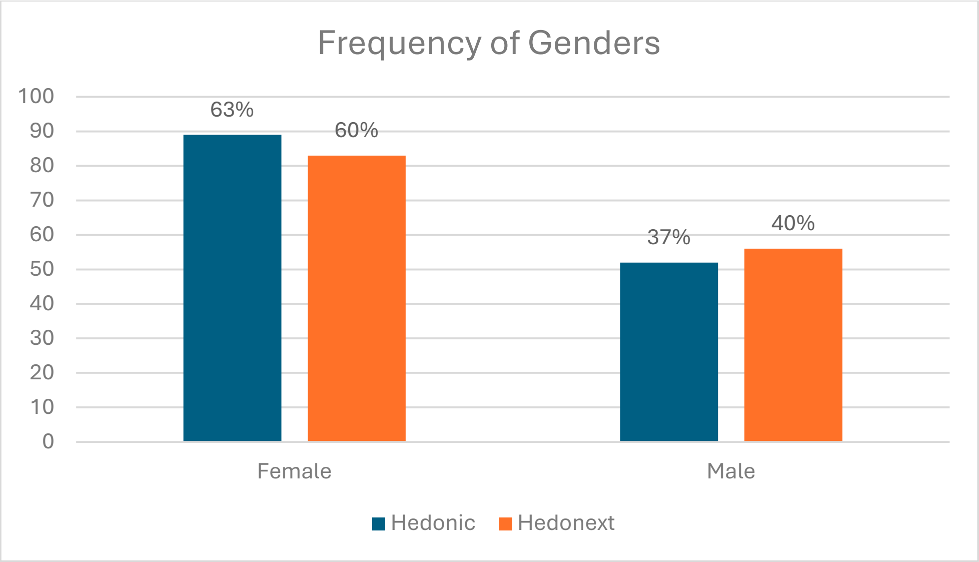

c. Ethnicity

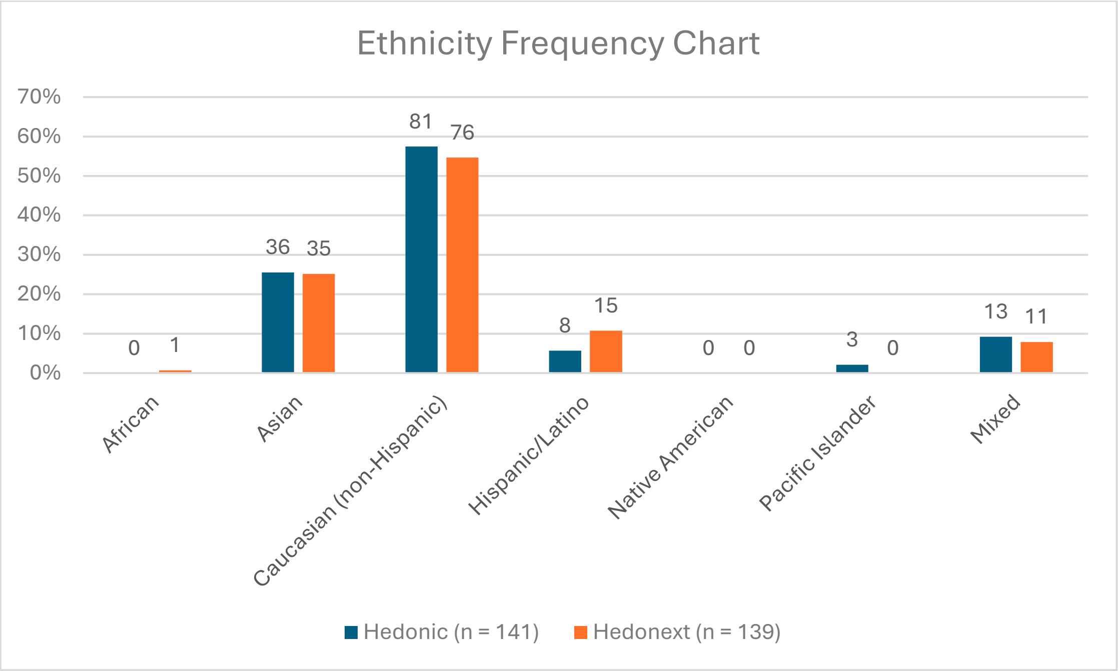

d. Education level

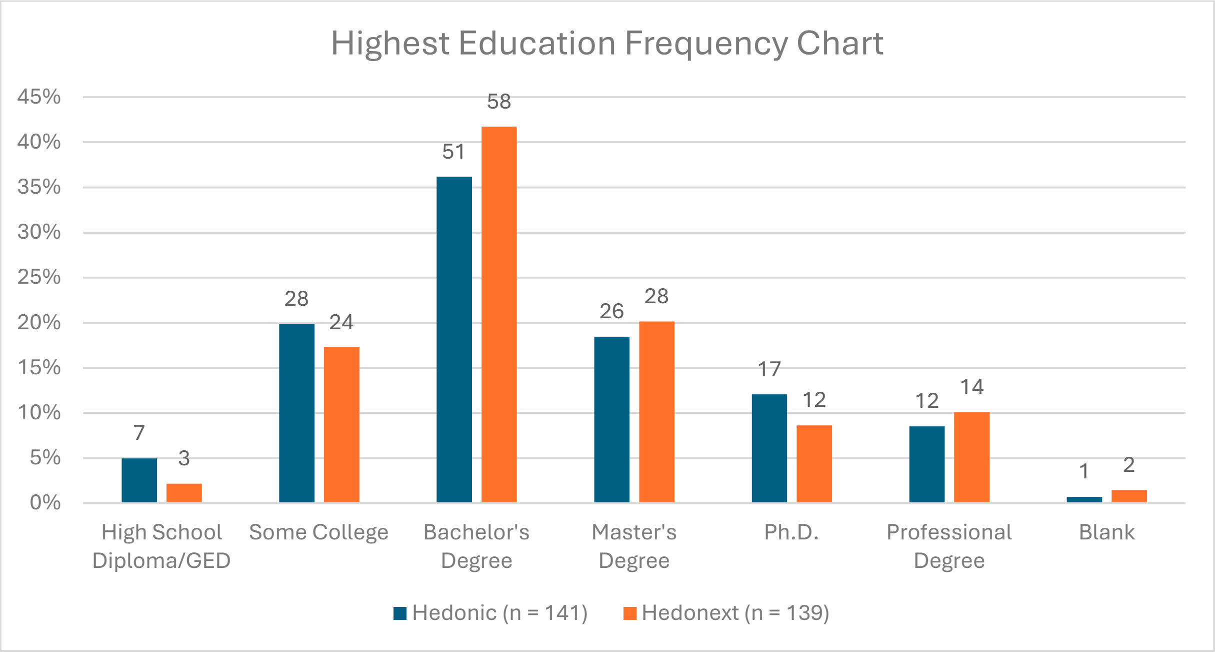

e. Household income

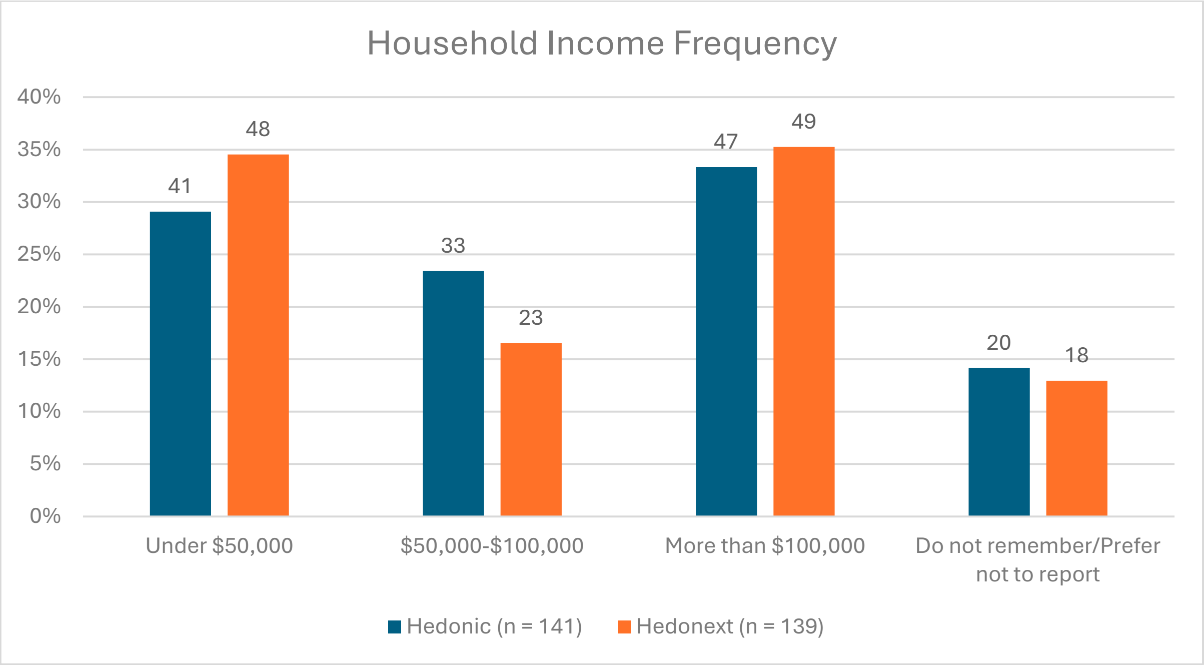

f. Olive oil consumption

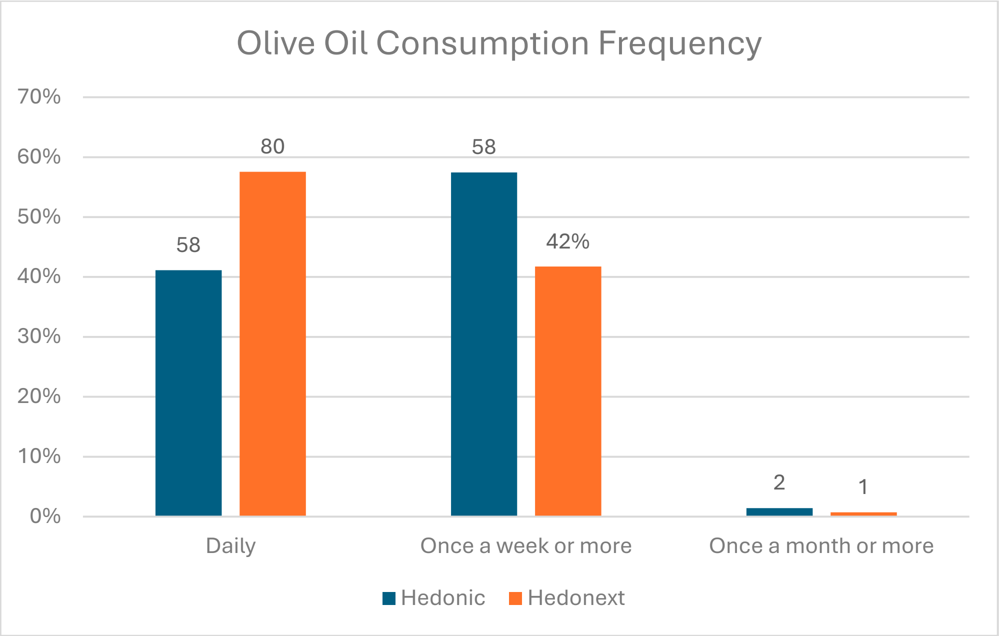

g. Type of olive oil bought

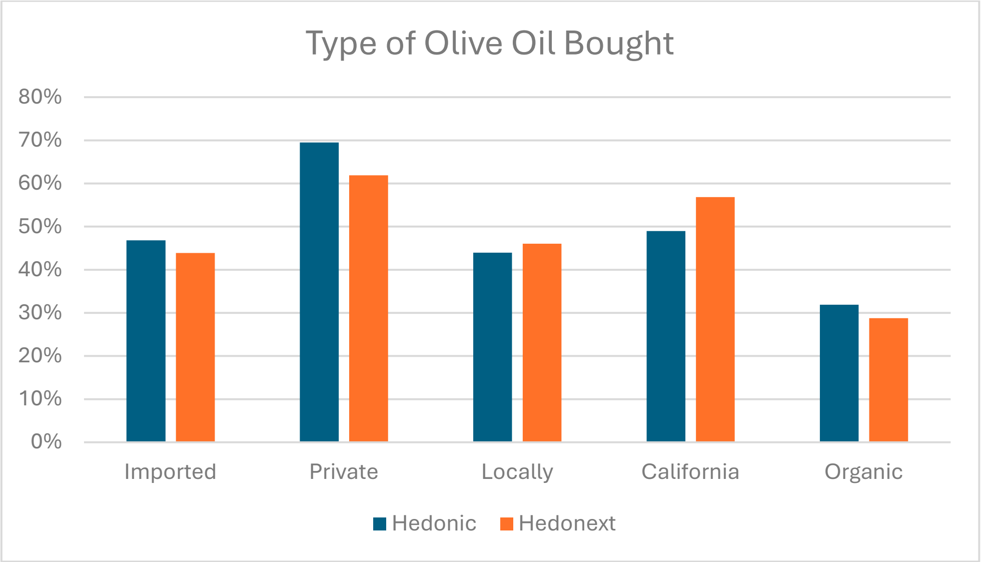

h. Typical olive oil use

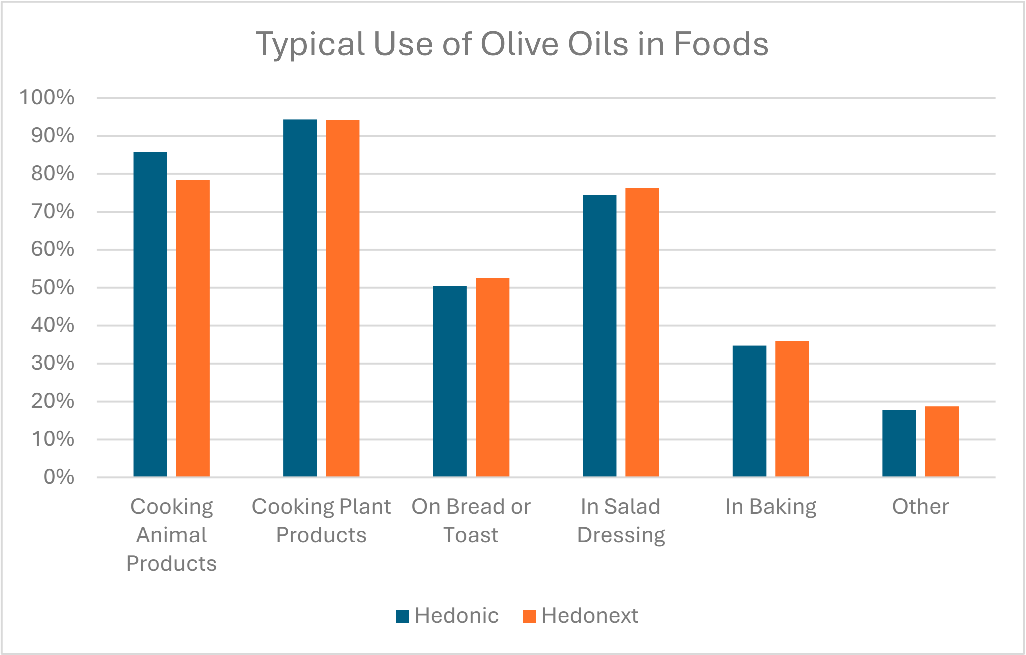

Supplement: Supplementary file 1 [file foods-15-00276-s001.zip › foods-4010957-supplementary.pdf]
